# Usefulness of Chest Ultrasonography in Predicting Diagnosis in Non-emergency Small Animal Patients With Lung Parenchymal and Pleural Disease

**DOI:** 10.3389/fvets.2020.616882

**Published:** 2020-12-18

**Authors:** Chung-Hui Lin, Pei-Ying Lo, Man-Cham Lam, Huey-Dong Wu

**Affiliations:** ^1^National Taiwan University Veterinary Hospital, National Taiwan University, Taipei, Taiwan; ^2^Graduate Institute of Veterinary Clinical Sciences, School of Veterinary Medicine, National Taiwan University, Taipei, Taiwan; ^3^Section of Respiratory Therapy, Department of Integrated Diagnostics and Therapeutics, National Taiwan University Hospital, National Taiwan University, Taipei, Taiwan

**Keywords:** cat, chest ultrasonography, dog, non-invasive clinical assessment, pulmonology, respiratory disease

## Abstract

Chest ultrasonography has become an indispensable tool for pulmonary specialists in human medicine, but its current use in dogs and cats is primarily for emergency. The diagnostic performances of various ultrasonographic features other than comet-tail artifacts are of limited information in veterinary literatures. Therefore, the aims of this retrospective study were to investigate ultrasonographic findings in feline and canine respiratory patients with lung parenchymal and pleural space diseases, and to assess how ultrasonographic features correspond to specific diagnoses. Sixty-five non-emergency cases with radiographically identified lung parenchymal and pleural space abnormalities were included. Medical records and ultrasound video clips were reviewed, and additional follow-up information was subsequently collected. Common findings such as comet-tail artifacts (87.7% of cases), consolidation (84.6%), and thickened/irregular pleura (69.2%) were not distinguishable for a specific diagnosis. The presence of nodular/mass-like lesion (OR = 212, *p* < 0.001) and consolidated lesion with heteroechogenicity (OR = 240, *p* < 0.001) was significantly associated with and strongly predictive of neoplasia after age, body weight and other sonographic findings were adjusted. The finding of nodular/mass-like lesion has the best diagnostic performance (AUC = 0.93) for neoplasia, with sensitivity of 91.7% and specificity of 93.6%. For predicting a diagnosis of pneumonia, although several sonographic features were found to be statistically associated with pneumonia, only a negative finding of nodular/mass-like lesion showed good diagnostic performance (AUC = 0.83, sensitivity 95.7%, specificity 71%). These findings demonstrate the value of chest ultrasonography in predicting diagnosis in non-emergency cases. The application of thoracic ultrasound in small animal respiratory patients as part of non-invasive assessment warrants further investigation.

## Introduction

Ultrasound has become a common diagnostic procedure in small animal clinical medicine over the past decades. Although the application of ultrasound on the thorax can be limited by aerated lung in normal individuals, acoustic window is created with the presence of pleural effusion and non-aerated lung in diseased patients (1–4). For dogs and cats with respiratory distress, point-of-care lung ultrasound is a clinically useful and easily available tool for the purpose of initial assessment in emergency room and intensive care unit (5–7). The presence/absence and characteristics of comet-tail artifacts (also known as lung rockets, ring-down artifacts, or B line) on lung ultrasound have currently been evaluated for diagnostic performance in canine and feline patients with various emergency conditions, including lung contusion, pneumothorax, cardiogenic pulmonary edema, and alveolar-interstitial syndrome (8–15).

Other sonographic features that have also been described as abnormal findings of thoracic ultrasound in dogs and cats include the presence of pleural effusion, subpleural nodule, pulmonary mass, consolidation, atelectasis, and ruptured diaphragm (1, 13, 15–17). The diagnostic value of ultrasound-guided fine-needle aspiration/biopsy of consolidated pulmonary lesions or thoracocentesis for effusion drainage has been reported in earlier studies and case reports (1, 16, 18, 19). A recent study focusing on coughing dogs reported that subpleural shred signs and nodule signs were more often present in dogs with bacterial pneumonia and pulmonary neoplasia, respectively (15). These findings imply that the utility of chest ultrasonography in small animal patients warrants further attention and investigations.

Chest ultrasonography has been extensively studied and clinically applied in patients with different kinds of respiratory problems in human medicine (4, 20). In patients with pleural effusion, it has been found that sonographic appearance of a complex, septated or echogenic effusion may imply an exudative process, whereas two-thirds of exudative effusions were shown to be anechoic (4). This indicates that the clinical decision based on sonographic findings should still be carefully evaluated through clinical studies. Nevertheless, ultrasound helps detect more pneumonia and other lung pathologies than thoracic radiography, and sonographic features of consolidated lung such as air/fluid bronchogram, vascular pattern, or the presence of comet-tail artifacts could aid in distinguishing pneumonia, pulmonary thromboembolism, lung cancer, or lung contusion (3, 4). For instance, sonographic features of consolidation with air bronchogram and an ill-defined margin were seen to be present in ~90% of pneumonia patients, whereas nodules with well-defined margin and the absence of air bronchogram were more commonly seen in patients with lung neoplasia (3, 21, 22). Overall, ultrasound has been considered as a useful modality to improve diagnostic and therapeutic capabilities of pulmonary specialists outside of the intensive care unit (23).

Data are limited in the current veterinary literatures regarding the diagnostic performances of various chest ultrasonographic abnormalities other than comet-tail artifacts in dogs and cats. Therefore, the objectives of this study were to review chest ultrasonographic findings in dogs and cats with radiographically identified lung parenchymal and pleural space abnormalities and to assess the diagnostic performance of various sonographic features corresponding to specific diagnoses.

## Materials and Methods

The present study was performed as a retrospective review of medical records and sonographic imaging files at a university teaching hospital. This study was reviewed and approved by the Research Ethical Committee (REC) of National Taiwan University Veterinary Hospital (Approval No: 000036) and the Institutional Animal Care and Use Committee (IACUC) of National Taiwan University (Approval No: NTU106-EL-00209). The case logs of canine and feline outpatients with respiratory disease and non-emergency visits were searched from February 2013 to December 2018. Animals with radiographically identified lung parenchymal and pleural space abnormalities and examined by thoracic ultrasound were reviewed. Cases were included only if real-time video clips of sonographic scans were available, and all videos were reviewed to ensure that the sonographic findings were properly addressed. The medical records of each patient were reviewed for signalment, physical examination findings, radiographic abnormalities, laboratory data, the reason for receiving chest ultrasonographic examination, any further diagnostic procedures, diagnosis, and follow-up information. The final diagnosis for retrospective analyses was determined based on a combination of cytological or histopathological evaluation, computed tomography imaging, echocardiography/cardiologic examinations, other laboratory findings, clinical follow-up, or necropsy. Additional follow-up information was continually collected until December 2019, either from the patient attending the follow-up visit or by the telephone interview.

Chest ultrasonography was performed either by an experienced attending clinician (CHL) or by residents under the supervision of the same attending clinician. Three ultrasound machines (EnVisor, Philips; MyLab50, Esaote; EPIQ 7, Philips) were available during the study period. Total gain, near- and far-field gains, depth, and focal point were adjusted to optimize image quality. A convex probe (5–8 or 3–9 MHz) was most often selected for scanning. Sector (3–8 or 4–12 MHz) or linear (7–15 MHz) probes were also available but occasionally used in these retrospective cases. The hair was clipped before scanning. The animals were positioned in sternal recumbent, standing, or sitting position if possible. Adequate acoustic windows were obtained through intercostal spaces, thoracic inlet, parasternal, or subcostal locations based on each case's condition and radiographic findings. Transverse imaging planes were preferable, but longitudinal planes were also used whenever suitable (1, 2, 4, 16, 23).

Abnormal findings on chest ultrasonography were predefined based on previous literatures (1, 3, 4, 8, 17, 24, 25). Sonographic imaging files in each case were reviewed for the presence or absence of the following abnormalities: pleural effusion, thickened or irregular pleura, comet-tail artifacts, lung atelectasis, consolidation, peripheral pulmonary nodule, mass-like lesion, and non-continuous diaphragm. Table 1 lists the features of abnormal thoracic ultrasound findings.

**Table 1:** Descriptions of the sonographic features of abnormal thoracic ultrasound findings predefined in this study.

Pleural effusion (24)	Presence of anechoic or echogenic fluid between visceral pleura and the parietal pleura
Anechoic	Echo-free fluid
Homogenous echogenic	Homogeneously echogenic fluid
Complex, non-septated	Heterogeneous echogenic material inside the anechoic effusions
Complex, septated	With fibrin strands or septa floating inside the effusions
Thickened or irregular pleura (4)	Presence of pleural thickening or irregularities of the pleural margins, instead of a smooth echogenic to hypoechoic line
Comet-tail artifacts (1, 8)	Vertical reverberation artifacts extended from the pulmonary-pleural interface to the far field
Consolidation (3, 4, 25)	An echoic or hypoechoic area with tissue-like echotexture and ill-defined margin at far field. With or without air bronchogram or fluid bronchogram
Focal consolidation	Involve focal or multifocal area
Partial consolidation	Involve partial but not the entire lung lobe
Lobar consolidation	Involve the entire lung lobe, with hepatization appearance
Air bronchogram	Multiple echogenic foci caused by residual air within alveoli and bronchi
Fluid bronchogram	Tubular branching structure with anechoic or hypoechoic lumen which has no blood flow detected by Doppler ultrasound
Atelectasis (4, 25)	Collapsed (reduced volume) lung lobe which can be identified as small triangular, echogenic structures, with or without air bronchogram
Peripheral pulmonary nodule(s) (25)	Small hypoechoic areas beneath the visceral pleura, with relatively smooth and regular margins
Mass-like lesion(s) (3, 25)	A solid mass-like area with relatively well-defined margins and variable echo textures
Non-continuous diaphragm (17, 25)	Discontinuity of the curvilinear echogenicity between the liver-diaphragm and lung, or look for evidence of abdominal viscera in the thorax

Statistical analyses were performed using statistical software (MedCalc 19.1; MedCalc Software bv, Ostend, Belgium; R 3.6.1; R Core Team 2019, Vienna, Austria). Data with continuous variables were reported as median and range. Chi-square test or Fisher's exact test was used to assess the association between each sonographic feature and a final diagnosis. The relationship between canine and feline species, age, gender, body weight, sonographic findings, and the diagnosis of neoplasia or pneumonia were analyzed by using logistic regression model, and the variables with *p* ≤ 0.25 in the univariable logistic regression model were selected into the multivariable logistic regression model. Penalized maximum likelihood estimation was used with logistic regression with a small number of frequency count or with the presence of empty cell frequency in 2 × 2 table to reduce bias in logistic regression in small samples (26). For sonographic characteristics that were likely to predict the diagnosis of neoplasia or pneumonia in multivariable logistic regression model, receiver operating characteristic curves (ROC) were constructed to assess the sensitivity and specificity of a particular sonographic finding.

## Results

The study population was comprised of 65 non-emergency visit cases with radiographically identified lung parenchymal and pleural space abnormalities during February 2013 to December 2018 and met with inclusion criteria of this study. Median (range) age of these 46 cats and 19 dogs was 10 (0.3–16) years and 11 (0.5–17) years, respectively. Median (range) body weight was 4.5 (1.1–7.4) kg in cats and 10.4 (1.2–30.9) kg in dogs. All the cases were received chest ultrasonographic examination because of the following reasons: to investigate the abnormal opacity on thoracic radiograph, to guide fine-needle aspiration of nodule/mass-like lesion, to assist thoracocentesis, or to evaluate the characteristics of pleural effusion.

The frequency of the presence of predefined abnormalities in 65 cases were as following: the most common finding was comet tail artifacts shown in 87.7% of cases, followed by consolidation in 84.6%, thickened or irregular pleura in 69.2%, nodule or mass-like lesion in 40%, pleural effusion in 35.4%, lung atelectasis in 21.5%, and non-continuous diaphragm in 4.6%.

The etiologies in 10 of 65 cases (15.4%) were undetermined and excluded from the subsequent analyses. Final diagnoses obtained in 55 cases (84.6%) (37 cats and 18 dogs) were included neoplasia in 23 of 55 (41.8%), pneumonia in 20 of 55 (36.4%), congestive heart failure in 3 of 55 (5.5%), chylothorax in 2 of 55 (3.6%), lung atelectasis caused by chronic lower airway disease in 2 of 55 (3.6%), and one case (1.8%) each with peritoneal-pericardial diaphragmatic hernia, lung parenchymal granuloma, concurrent pneumonia with bullae lung disease, concurrent pneumonia with cardiogenic pulmonary edema, or concurrent pneumonia with neoplasia.

A final diagnosis of neoplasia was made based on the supportive evidence from cytology or histopathology (fine-needle aspiration, biopsy, or necropsy) in 15/24 of cases, consisting of four adenocarcinomas, three tumors of epithelial origin, two carcinomas, two tumors of an uncertain cell type, one squamous cell carcinoma, one neuroendocrine carcinoma, and one metastasis of a mammary gland tumor. If cytological or histopathological evaluation was unavailable (9/24 cases), clinical diagnosis of neoplasia was accepted if it was unlikely to be other etiologies based on the overall clinical presentation (e. g., high likelihood of neoplastic lesion suggested by computed tomography findings; metastasis from previously diagnosed neoplasia supported by imaging findings; history, other clinical data and subsequent outcome highly suggestive of malignancy; presence of multiorgan infiltration). The number of primary neoplasia (18/24) cases was higher than that of metastatic neoplasia (4/24). The primary tumor in two cases with neoplastic lesions on more than one location was indistinguishable.

Diagnosis of pneumonia was given if showed adequate treatment response to antimicrobial agents (16/23 cases). In cases with inadequate response to antimicrobial agents (7/23), the diagnosis of pneumonia was made based on findings by cytology, microbiological tests, computed tomography, or necropsy. A congestive heart failure status was confirmed by echocardiography and other necessary diagnostics. Chylothorax was diagnosed by comparing triglyceride and cholesterol concentrations in pleural effusion with serum values. Lung atelectasis caused by chronic lower airway disease was diagnosed based on medical history and apparent volume reduction of the lung lobe on imaging findings. Hernia was confirmed by computed tomography and surgery. Lung parenchymal granuloma was confirmed by histopathology. Bullae lung disease was diagnosed by computed tomography.

### Neoplasia-Associated Sonographic Characteristics

**Figure 1:**
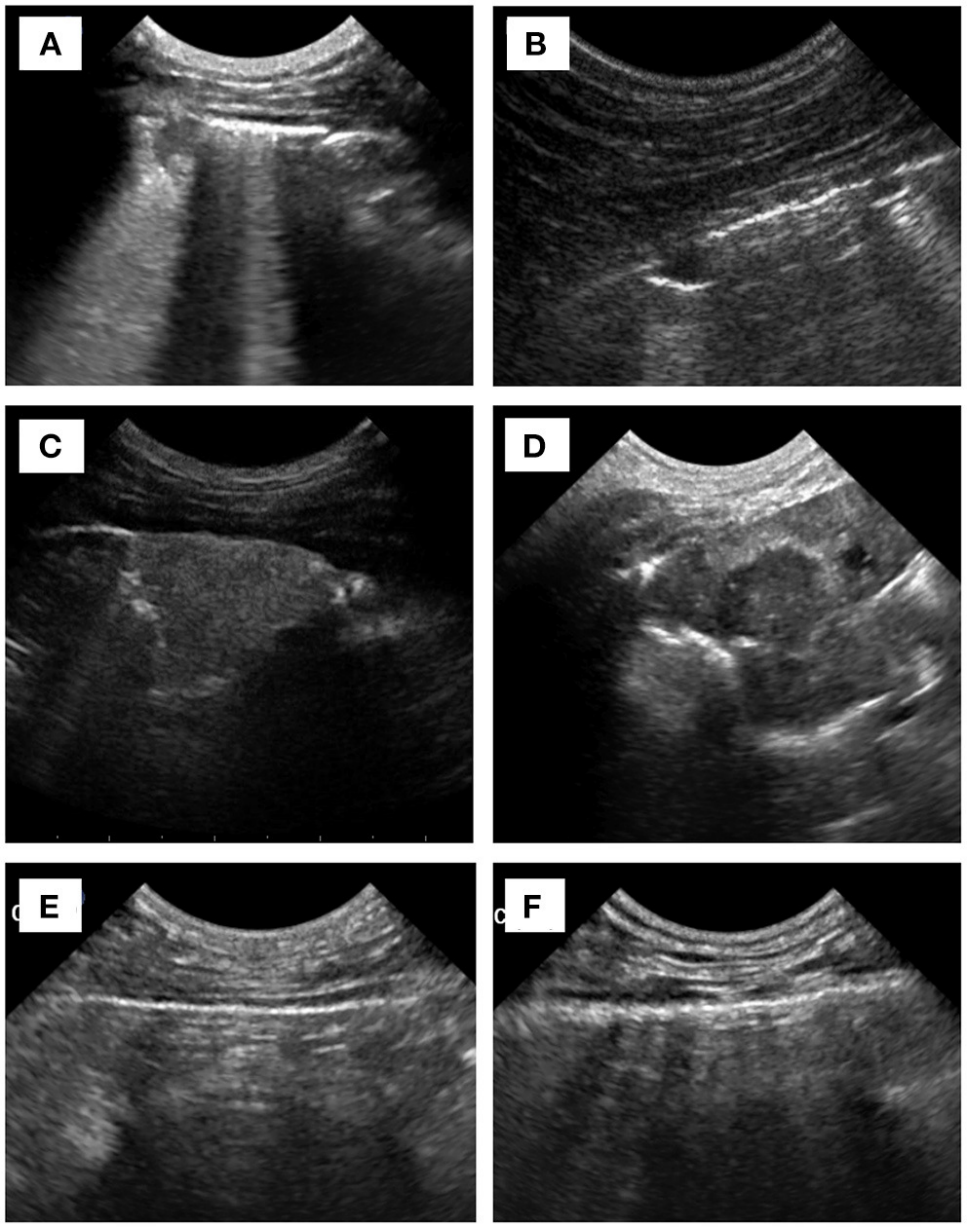
Sonographic images of **(A)** focal consolidation vs. **(B)** nodular-like lesion, **(C)** consolidation with homoechogenicity vs. **(D)** heteroechogenicity, and **(E)** normal pleura vs. **(F)** thickened/irregular pleura.

Nodular or mass-like lesion (*p* < 0.001) and consolidated lesion with heteroechogenicity (*p* = 0.002) were found to be significantly associated with the diagnosis of neoplasia (Table 2, Figure 1). The presence of pleural effusion, comet-tail artifacts, thickened or irregular pleura, consolidation, and atelectasis were not associated with neoplasia. Species and gender were not statistically associated with neoplasia. Animals with older age were found to be possible risk factors for the diagnosis of neoplasia in univariable logistic regression analyses considering both signalment and sonographic characteristics. “Nodular or mass-like lesion” and “consolidated lesion with heteroechogenicity” were found to be recognized as significant predictors of neoplasia when age, body weight, and other sonographic findings were adjusted (Table 3).

**Table 2:** Sonographic characteristics in all 65 cases and in cases with final diagnosis as neoplasia or pneumonia.

**Sonographic findings**	**All (*n* = 65)**	**Neoplasia (*n* = 24)^a^**	**Pneumonia (*n* = 23)^b^**
Pleural effusion	35.4% (23/65)	33.3% (8/24)	26.1% (6/23)
Anechoic	26.2% (17/65)	20.8% (5/24)	21.7% (5/23)
Homogenous echogenic	16.9% (11/65)	20.8% (5/24)	8.7% (2/23)
Complex, non-septated	6.2% (4/65)	4.2% (1/24)	4.3% (1/23)
Complex, septated	4.6% (3/65)	0.0% (0/24)	4.3% (1/23)
Thickened or irregular pleura	69.2% (45/65)	66.7% (16/24)	87.0% (20/23)^d^
Comet tail artifacts	87.7% (57/65)	91.7% (22/24)	100.0% (23/23)
Consolidation	84.6% (55/65)	87.5% (21/24)	95.7% (22/23)^d^
Focal consolidation	69.2% (45/65)	70.8% (17/24)	87.0% (20/23)^d^
Partial consolidation	50.8% (33/65)	41.7% (10/24)	73.9% (17/23)^d^
Lobar consolidation	16.9% (11/65)	12.5% (3/24)	21.7% (5/23)
Atelectasis	21.5% (14/65)	20.8% (5/24)	8.7% (2/23)
Nodular or mass-like lesion	40.0% (26/65)	91.7% (22/24)^c^	4.3% (1/23)^d^
Non-continuous diaphragm	4.6% (3/65)	4.2% (1/24)	0.0% (0/23)

a *Cases here included: 23 cases with a final diagnosis of neoplasia and 1 case with concurrent neoplasia and other diseases*.

b *Cases here included: 20 cases with a final diagnosis of pneumonia and 3 cases with concurrent pneumonia and other diseases*.

c *Significant difference (p < 0.05) between animals with and without neoplasia in chi-square or Fisher's exact test*.

d *Significant difference (p < 0.05) between animals with and without pneumonia in chi-square or Fisher's exact test*.

**Table 3:** Univariable and multivariable logistic regression analyses of signalment and sonographic findings predictive of neoplasia.

**Variable**	**Univariable**		**Multivariable^a^**	
	**Crude OR (95% CI)**	***p***	**Adjusted OR (95% CI)**	***p***
**SIGNALMENT**				
Species (dog/cat)	1.75 (0.57–5.36)	0.33	–	–
Sex (male/female)	1.56 (0.53–4.63)	0.42	–	–
Neutered status (yes/no)	1.68 (0.37–7.55)	0.50	–	–
Age (years)	**1.29 (1.07–1.56)**	**0.008**	1.39 (0.94–3.18)	0.11
Body weight (kg)	1.07 (0.97–1.18)	0.18	1.04 (0.89–1.40)	0.70
**SONOGRAPHIC FINDINGS**				
Pleural effusion	0.91 (0.30–2.80)	0.87	–	–
Anechoic	0.64 (0.18–2.25)	0.49	–	–
Homogenous echogenic	2.46 (0.52–11.5)	0.25	4.38 (0.15–1287)	0.46
Complex, non-septated	0.63 (0.05–7.39)	0.71	–	–
Complex, septated	0.24 (0.002–3.14)	0.30	–	–
Thickened or irregular pleura	0.70 (0.22–2.24)	0.54	–	–
Comet-tail artifacts	1.18 (0.18–7.68)	0.86	–	–
Consolidation	2.04 (0.47–8.91)	0.34	–	–
With heteroechogenicity	**27 (2.98–3583)**	**0.001**	**240 (7.62–70329)**	**0.001**
Atelectasis	0.76 (0.21–2.70)	0.67	–	–
Nodular or mass-like lesion	**106 (21–878)**	**<0.001**	**212 (21–31388)**	**<0.001**
Non-continuous diaphragm	1.30 (0.08–22.0)	0.85	–	–

*Bolded values indicating two-tailed p < 0.05*.

a *The variables with p >0.25 in the univariable logistic regression model or with collinearity property were not selected into the multivariable logistic regression model*.

The diagnostic performance of a finding of nodular or mass-like lesion by ROC curve showed an area under the ROC curve (AUC) was 0.93 [95% confidence interval [CI]: 0.82–0.98], with sensitivity of 91.7% and specificity of 93.6%. The AUC for consolidated lesion with heteroechogenicity was 0.65 (95% CI: 0.51–0.77), with sensitivity of 29.2% and specificity of 100%.

### Pneumonia-Associated Sonographic Characteristics

The presence of thickened or irregular pleura (*p* = 0.034), consolidation (*p* = 0.032), and the absence of nodular or mass-like lesion (*p* < 0.001) were significantly associated with the diagnosis of pneumonia (Table 2). The presence of pleural effusion, comet-tail artifacts, atelectasis, and echogenicity features of consolidated lesion were not associated with pneumonia. Species and gender were not statistically associated with pneumonia. Animals with younger age were also found to be possible risk factors for pneumonia in univariable logistic regression analyses, taking both signalment and sonographic characteristics into consideration. “The presence of consolidation” and “a negative finding for nodular/mass-like lesion” were found to be the significant predictors for the diagnosis of pneumonia after age, body weight, and other sonographic findings were adjusted (Table 4).

**Table 4:** Univariable and multivariable logistic regression analyses of signalment and sonographic findings predictive of pneumonia.

**Variable**	**Univariable**		**Multivariable^a^**	
	**Crude OR (95% CI)**	***p***	**Adjusted OR (95% CI)**	***p***
**SIGNALMENT**				
Species (dog/cat)	0.69 (0.22–2.18)	0.53	–	–
Sex (male/female)	1.07 (0.36–3.17)	0.90	–	–
Neutered status (yes/no)	0.53 (0.13–2.26)	0.39	–	–
Age (years)	**0.83 (0.71–0.97)**	**0.02**	0.80 (0.54–1.04)	0.10
Body weight (kg)	0.98 (0.89–1.07)	0.66	–	–
**SONOGRAPHIC FINDINGS**				
Pleural effusion	0.49 (0.15–1.58)	0.23	1.03 (0.11–11.5)	0.98
Anechoic	0.68 (0.19–2.39)	0.55	–	–
Homogenous echogenic	0.40 (0.07–2.18)	0.29	–	–
Complex, non-septated	0.66 (0.06–7.74)	0.74	–	–
Complex, septated	1.36 (0.08–23.0)	0.83	–	–
Thickened or irregular pleura	**4.81 (1.18–19.7)**	**0.03**	13.8 (0.67–916)	0.093
Comet-tail artifacts	**9.75 (1.02–1306)**	**0.048**	0.31 (0.002–121)	0.66
Consolidation	**9.00 (1.05–77.2)**	**0.045**	**19.6 (1.67–469)**	**0.018**
With heteroechogenicity	0.19 (0.02–1.70)	0.14	–	–
Atelectasis	0.20 (0.04–1.03)	0.054	0.49 (0.04–5.28)	0.55
Nodular or mass-like lesion	**0.02 (0.002–0.16)**	**<0.001**	**0.02 (0.001–0.13)**	**<0.001**
Non-continuous diaphragm	0.25 (0.002–3.28)	0.32	–	–

*Bolded values indicating two-tailed p < 0.05*.

a *The variables with p >0.25 in the univariable logistic regression model or with collinearity property were not selected into the multivariable logistic regression model*.

The negative finding of nodular/mass-like lesion by ROC curve showed an AUC as 0.83 (95% CI: 0.71–0.92), with sensitivity of 95.7% and specificity of 71%. The diagnostic performance of comet-tail artifacts (AUC 0.74, sensitivity 46.9%, specificity 100.0%), thickened or irregular pleura (AUC 0.67, sensitivity 52.6%, specificity 81.3%), and consolidation (AUC 0.70, sensitivity 50.0%, specificity 90.0%) were found to be poorer than a negative finding of nodular/mass-like lesion.

## Discussion

Results of the present study provide information on diagnostic performance of various imaging features of thoracic ultrasound in small animal patients. Common findings such as comet-tail artifacts, consolidation, and thickened/irregular pleura are not distinguishable for a specific diagnosis. Although a final diagnosis cannot be relied solely on the sonographic appearance, some characteristics on chest ultrasonography could help predict the etiology. The presence of “nodular/mass-like lesion” and “consolidated lesion with heteroechogenicity” could predict neoplasia, and the finding of “nodular/mass-like lesion” has the best diagnostic performance for this. The presence of “consolidation” along with “a negative finding of nodular/mass-like lesion” after careful scanning helps predict a diagnosis of pneumonia.

All cases in this study had shown certain abnormalities on thoracic radiographs, and therefore they underwent subsequent evaluation. Thoracic radiography is usually the first approach in clinical patients with lung parenchymal abnormalities. However, the information from radiography can be lessened by the presence of parapneumonic effusion or heavy alveolar-interstitial infiltration adjacent to neoplastic lesions (3, 4, 21, 23, 24), which likely to obscure the underlying etiology. Although computed tomography scan can help clarify the anatomical relationship between lesions and surrounding structures, the high cost and the common need for general anesthesia in small animal patients may limit its use. Ultrasonographic evaluation allows safe, low cost, radiation-free, and easily reproducible imaging modality in demonstrating varied etiologies in human patients with lung parenchymal abnormalities, such as pneumonia and cancer infiltration manifesting as lung consolidation (3, 20, 23). Similarly, the present study also found diverse etiologies and the value of applying chest ultrasonography as a part of diagnostic assessment in small animal patients with lung parenchymal and pleural space abnormalities.

Thoracic ultrasound accompanied by guided fine-needle aspiration or biopsy in dogs and cats with non-cardiac intrathoracic lesions or mass lesions was described as a valuable tool in previous studies (1, 16). In contrast, not all cases in our study received ultrasound-guided sampling for variable reasons (e.g., owners declined ultrasound-guided procedures, having only small amount of effusion, clinicians estimated to have difficulty in manipulating some of the patients, etc.). The reasons of not receiving ultrasound-guided procedures are sometimes inevitable clinical reality that all clinicians may face when handling small animal patients. This highlights the need to evaluate sonographic characteristics that will help predict the underlying etiology.

It was reported in a previous study that inflammatory and neoplastic lesions could not be distinguished by ultrasonographic features (16); however, relatively few animals (16 dogs and three cats) included in that previous work and the lack of statistical analysis may lead to the inconclusive observation. In another earlier study with larger numbers of dogs and cats, most cases enrolled were of neoplastic etiology (47/75), and only one single case diagnosed with eosinophilic lung disease was found to be of inflammatory etiology (1). Therefore, it could be assumed that general ultrasonographic characteristics of pneumonia and the associated diagnostic performance were less clear in dogs and cats. On the contrary, in a recent systematic review and meta-analysis of human patients it was reported that pneumonia can be accurately diagnosed by lung ultrasonography (20). Blurred margins of consolidation with variable air bronchogram seem to be the most common finding among human patients with pneumonia (4, 21, 27). Although this feature also seemed to be commonly found in small animal patients with pneumonia, our results showed the diagnostic performance of a negative finding of nodular/mass-like lesion was better than the presence of consolidation. Moreover, inflammatory consolidation is also likely to be present at the peripheral of a neoplasm (2, 16). In considering the severity of lesions on lung ultrasound can be underestimated without the entire thorax scanning (28), a more extensive scanning of the thorax is necessary before presuming an ultrasonographic diagnosis of pneumonia in small animal patients.

A neoplastic lesion usually shows well-defined and smooth borders in contrast to ill-defined or blurred margins of consolidation (1, 3). Otherwise, in a previous study of 26 cats and 49 dogs, the echo texture of all neoplastic lesions was described as homogeneously hypoechoic (1). In our study, the presence of a nodular or mass-like lesion (with relatively well-defined and smooth margin as predefined) was shown to be a strong predictor for the diagnosis of neoplasia. However, consolidated lesion with heteroechogenicity was also significantly associated with neoplasia, though with a much lower sensitivity. Conversely, the feature of a heterogeneous echogenic pattern was not found to be predictive of malignancy in a prospective study on human patients (22). Considering this and the fact that some neoplastic lesions were also present as hyperechoic (3, 22), the feature of margin rather than the echo texture of a lesion might be more distinguished for the diagnosis of malignancy.

Pleural effusion in cats was commonly reported to be associated with neoplasia, and its presence usually indicates guarded prognosis and short survival (29–32). It is worth evaluating the sonographic appearance of pleural effusion for predicting neoplastic etiology. Nevertheless, neither the presence of pleural effusion nor the echogenic characteristics of the effusion was statistically associated with neoplastic etiology in the present study. It is not surprised that pleural effusion is not specific for a certain diagnosis, while it could also be found in patients with congestive heart failure, pneumonia, or other pleural disorder. Therefore, the occurrence of pleural effusion and the echogenic characteristics of the effusion were not predictive of malignancy based on our results, but pleural effusion could imply poor prognosis in cats with sonographic evidence of a nodular or mass-like lesion suggestive of neoplasia.

Comet-tail artifacts, shown in 88% of cases, was the most prevalent finding among all sonographic abnormalities in the present study. The application of using the severity of comet-tail artifacts, so-called B line on the point-of-care lung ultrasound, to help determine the presence of cardiogenic pulmonary edema in dogs and cats has been evaluated in recent years (11–13, 15). The generation of comet-tail artifacts is caused by the impedance gradient between fluid/tissue and surrounding air; thus, any alveolar or interstitial pulmonary pathology can result in this type of artifacts (14). Consequently, the presence of comet-tail artifacts is probably the least specific finding for aiding diagnosis, but it is supposed to be sensitive and more suitable for use in emergency lung ultrasound.

In this study, convex probes were primarily used, and sector and linear probes were occasionally used, depending on the operator's preference, equipment availability, and body size of the animals. Various types of ultrasound probes were used in previous studies in dogs and cats, including sector (7–7.5 MHz) (1, 16, 17), convex (4–10 MHz) (7, 13), linear (2.4–15 MHz) (9–11, 16), and curvilinear (5–8 MHz) (8, 12–15) probes. The advantage of using a linear probe is the increase in the resolution of near-field structures, which caused impaired penetration of lesions at deeper location in a consolidated lung or within a large effusion (4, 23). For smaller-sized patients with restricted intercostal spaces, convex or sector probes provide smaller footprint and fit better for evaluation (2, 4, 23). Probe selection should depend on the lesion of interest and imaging optimization and thus be determined on a case basis. In general, convex probes with frequency of 5–8 or 3–9 MHz work well for most study animals.

The retrospective study design brings about unavoidable limitations. First, the scanning protocol was probably inconsistent in all cases; thus, some abnormalities were likely not found or recorded, leading to a bias in the analysis of the results. Furthermore, if a lesion was not located peripherally and no effusion can provide adequate acoustic window, ultrasonography would not be considered initially, which could lead to information bias. Moreover, as in all ultrasound techniques, intra- and inter-operator variability may affect diagnosis. Otherwise, not all cases diagnosed as neoplasia had undergone cytological or histopathological evaluation. Although the neoplastic etiology was supported by other clinical features such as computed tomography findings, the lack of gold standard diagnostics might result in misdiagnosis. Second, the study population in our work consisted of respiratory patients with non-emergency visit. It must be brought in mind that differences between emergency and non-emergency cases may exist and should be taken into consideration before applying the findings in this study. Finally, in the field of human chest ultrasonography, other methodologies such as Doppler characteristics that can aid in diagnosis are available, but these applications were not included in this study because of infrequent use in the present cases.

In conclusion, the findings in the present study provide information about sonographic features that help predict the underlying etiology of lung parenchymal and pleural space abnormalities in small animal patients. Although a diagnosis should not be based merely on the sonographic appearance, ultrasound can be used more often on small animal patients with respiratory diseases as a part of evaluation to guide further diagnostics. Our findings highlighted the importance of entire thoracic scanning for making clinical decision. If consolidation is present and pneumonia is clinically suspected, a thorough scanning for “a negative finding of nodular/mass-like lesion” can support the clinical diagnosis of pneumonia. The presence of neoplasia is highly suggestive if “nodular or mass-like lesion” and “consolidated lesion with heteroechogenicity” are noted. Future prospective investigation can be planned on the basis of this study to broaden the understanding of applying this non-invasive and easily available tool in small animal respiratory medicine.

## Data Availability Statement

The data sets for this article are not publicly available because they contain clinical patient data and the animals might be identifiable. The datasets generated for this study are available on request to the corresponding author.

## Ethics Statement

This study was reviewed and approved by the Research Ethical Committee (REC) of National Taiwan University Veterinary Hospital (Approval No: 000036) and the Institutional Animal Care and Use Committee (IACUC) of National Taiwan University (Approval No: NTU106-EL-00209). Formal written informed consent is not required for retrospective review. No identifiable detail of the individual is included in the manuscript and figure.

## Author Contributions

C-HL contributed to conception, study design, collection of the samples and data, data analysis, and preparation of the manuscript. P-YL contributed to collection of the samples and data and preparation of the manuscript. M-CL contributed to collection of the data and part of data analysis. H-DW contributed conception and study design. All authors contributed to the article and approved the submitted version.

## Conflict of Interest

The authors declare that the research was conducted in the absence of any commercial or financial relationships that could be construed as a potential conflict of interest.

## References

[1] Reichle JK Wisner ER. Non-cardiac thoracic ultrasound in 75 feline and canine patients. Vet Radiol Ultrasound. (2000) 41:154–62. 10.1111/j.1740-8261.2000.tb01470.x 10779076

[2] Larson MM. Ultrasound of the thorax (noncardiac). Vet Clin North Am Small Anim Pract. (2009) 39:733–45. 10.1016/j.cvsm.2009.04.006 19531398

[3] Yang PC Luh KT Chang DB Yu CJ Kuo SH Wu HD. Ultrasonographic evaluation of pulmonary consolidation. Am Rev Respir Dis. (1992) 146:757–62. 10.1164/ajrccm/146.3.757 1519859

[4] Dietrich CF Mathis G Cui XW Ignee A Hocke M Hirche TO. Ultrasound of the pleurae and lungs. Ultrasound Med Biol. (2015) 41:351–65. 10.1016/j.ultrasmedbio.2014.10.002 25592455

[5] Boysen SR Lisciandro GR. The use of ultrasound for dogs and cats in the emergency room AFAST and TFAST. Vet Clin North Am Small Anim Pract. (2013) 43:773–97. 10.1016/j.cvsm.2013.03.011 23747260

[6] Lisciandro GR. Abdominal and thoracic focused assessment with sonography for trauma, triage, andmonitoring in small animals. J Vet Emerg Crit Care. (2011) 21:104–22. 10.1111/j.1476-4431.2011.00626.x 21463438

[7] Lisciandro GR Fosgate GT Fulton RM. Frequency and number of ultrasound lung rockets (B- lines) using a regionally based lung ultrasound examination named vet blue (veterinary bedside lung ultrasound exam) in dogs with radiographically normal lung findings. Vet Radiol Ultrasound. (2014) 55:315–22. 10.1111/vru.12122 24382172

[8] Lisciandro GR Lagutchik MS Mann KA Voges AK Fosgate GT Tiller EG Evaluation of a thoracic focused assessment with sonography for trauma (TFAST) protocol to detect pneumothorax and concurrent thoracic injury in 145 traumatized dogs. J Vet Emerg Crit Care. (2008) 18:258–69. 10.1111/j.1476-4431.2008.00312.x

[9] Louvet A Bourgeois JM Lung ring-down artifact as a sign of pulmonary alveolar-interstitial disease. Vet Radiol Ultrasound. (2008) 49:374–7. 10.1111/j.1740-8261.2008.00384.x 18720771

[10] Rademacher N Pariaut R Pate J Saelinger C Kearney MT Gaschen L. Transthoracic lung ultrasound in normal dogs and dogs with cardiogenic pulmonary edema: a pilot study. Vet Radiol Ultrasound. (2014) 55:447–52. 10.1111/vru.12151 24620777

[11] Vezzosi T Mannucci T Pistoresi A Toma F Tognetti R Zini E. Assessment of lung ultrasound B-lines in dogs with different stages of chronic valvular heart disease. J Vet Intern Med. (2017) 31:700–4. 10.1111/jvim.14692 28370336 PMC5435052

[12] Ward JL Lisciandro GR Keene BW Tou SP DeFrancesco TC. Accuracy of point-of-care lung ultrasonography for the diagnosis of cardiogenic pulmonary edema in dogs and cats with acute dyspnea. J Am Vet Med Assoc. (2017) 250:666–75. 10.2460/javma.250.6.666 28263112

[13] Ward JL Lisciandro GR Ware WA Viall AK Aona BD Kurtz KA. Evaluation of point-of-care thoracic ultrasound and NT-proBNP for the diagnosis of congestive heart failure in cats with respiratory distress. J Vet Intern Med. (2018) 32:1530–40. 10.1111/jvim.15246 30216579 PMC6189386

[14] Ward JL Lisciandro GR DeFrancesco TC. Distribution of alveolar-interstitial syndrome in dogs and cats with respiratory distress as assessed by lung ultrasound vs. thoracic radiographs. J Vet Emerg Crit Care. (2018) 28:415–28. 10.1111/vec.12750 30075063

[15] Ward JL Lisciandro GR Ware WA Miles KG Viall AK DeFrancesco TC. Lung ultrasonography findings in dogs with various underlying causes of cough. J Am Vet Med Assoc. (2019) 255:574–83. 10.2460/javma.255.5.574 31429645

[16] Wood EF O'Brien RT Young KM. Ultrasound-guided fine-needle aspiration of focal parenchymal lesions of the lung in dogs and cats. J Vet Intern Med. (1998) 12:338–42. 10.1111/j.1939-1676.1998.tb02132.x 9773409

[17] Spattini G Rossi F Vignoli M Lamb CR. Use of ultrasound to diagnose diaphragmatic rupture in dogs and cats. Vet Radiol Ultrasound. (2003) 44:226–30. 10.1111/j.1740-8261.2003.tb01276.x 12718361

[18] Lynn A Dockins JM Kuehn NF Kerstetter KK Gardiner D. Caudal mediastinal thyroglossal duct cyst in a cat. J Small Anim Pract. (2009) 50:147–50. 10.1111/j.1748-5827.2008.00702.x 19261086

[19] Ho JC Chen HW Lin CH Hu KC. Fluid colour sign on chest ultrasonography in a cat with exudate pleural effusion and pleuropneumonia. J Small Anim Pract. (2019) 60:518. 10.1111/jsap.13043 31257592 PMC7166510

[20] Llamas-Alvarez AM Tenza-Lozano EM Latour-Perez J. Accuracy of lung ultrasonography in the diagnosis of pneumonia in adults: systematic review and meta-analysis. Chest. (2017) 151:374–82. 10.1016/j.chest.2016.10.039 27818332

[21] Gehmacher O Mathis G Kopf A Scheier M. Ultrasound imaging of pneumonia. Ultrasound Med Biol. (1995) 21:1119–22. 10.1016/0301-5629(95)02003-9 8849826

[22] Bugalho A Ferreira D Dias SS Schuhmann M Branco JC Marques Gomes MJ. The diagnostic value of transthoracic ultrasonographic features in predicting malignancy in undiagnosed pleural effusions: a prospective observational study. Respiration. (2014) 87:270–8. 10.1159/000357266 24480900

[23] Koenig SJ Narasimhan M Mayo PH. Thoracic ultrasonography for the pulmonary specialist. Chest. (2011) 140:1332–41. 10.1378/chest.11-0348 22045878

[24] Yang PC Luh KT Chang DB Wu HD Yu CJ Kuo SH. Value of sonography in determining the nature of pleural effusion: analysis of 320 cases. AJR Am J Roentgenol. (1992) 159:29–33. 10.2214/ajr.159.1.1609716 1609716

[25] Neelis DA Mattoon JS Nyland TG Thorax. In: Mattoon JS Nyland TG editors. Small Animal Diagnostic Ultrasound. 3rd Edn Missouri: Elsevier (2015). 10.1016/B978-1-4160-4867-1.00007-6

[26] Firth D Bias reduction of maximum-likelihood-estimates. Biometrika. (1993) 80:27–38. 10.1093/biomet/80.1.27

[27] Cortellaro F Colombo S Coen D Duca PG. Lung ultrasound is an accurate diagnostic tool for the diagnosis of pneumonia in the emergency department. Emerg Med J. (2012) 29:19–23. 10.1136/emj.2010.101584 21030550

[28] Lam MC Lo PY Wu HD Lin CH Lung ultrasound findings in dogs using a regionally based protocol (Vet BLUE) vs. entire thorax scanning. In: Congress Proceedings of the 29th European College of Veterinary Internal Medicine Congress 2019. Milano, Italy J Vet Intern Med (2020). p. 413.

[29] Davies C Forrester SD Pleural effusion in cats: 82 cases (1987 to 1995). J Small Anim Pract. (1996) 37:217–24. 10.1111/j.1748-5827.1996.tb01772.x 8736226

[30] Nunley J Sutton J Culp W Wilson D Coleman K Demianiuk R. Primary pulmonary neoplasia in cats: assessment of computed tomography findings and survival. J Small Anim Pract. (2015) 56:651–6. 10.1111/jsap.12401 26420583

[31] Konig A Hartmann K Mueller RS Wess G Schulz BS. Retrospective analysis of pleural effusion in cats. J Feline Med Surg. (2019) 21:1102–10. 10.1177/1098612X18816489 30554552 PMC10814271

[32] Ruiz MD Vessieres F Ragetly GR Hernandez JL. Characterization of and factors associated with causes of pleural effusion in cats. J Am Vet Med Assoc. (2018) 253:181–7. 10.2460/javma.253.2.181 29963947

